# Metadynamics studies of crystal nucleation

**DOI:** 10.1107/S2052252514027626

**Published:** 2015-02-10

**Authors:** Federico Giberti, Matteo Salvalaglio, Michele Parrinello

**Affiliations:** a Department of Chemistry and Applied Biosciences, ETH Zurich, CH-8092 Zurich, Switzerland; b Facoltá di informatica, Istituto di Scienze Computazionali, Universitá della Svizzera Italiana, CH-6900 Lugano, Switzerland; c ETH Zurich, Institute of Process Engineering, Soneggstrasse 3, CH-8092 Zurich, Switzerland

**Keywords:** crystallization, nucleation, molecular modelling, enhanced sampling, metadynamics

## Abstract

Crystallization processes are characterized by activated events and long timescales. These characteristics prevent standard molecular dynamics techniques from being efficiently used for the direct investigation of processes such as nucleation. This short review provides an overview on the use of metadynamics, a state-of-the-art enhanced sampling technique, for the simulation of phase transitions involving the production of a crystalline solid. In particular the principles of metadynamics are outlined, several order parameters are described that have been or could be used in conjunction with metadynamics to sample nucleation events and then an overview is given of recent metadynamics results in the field of crystal nucleation.

## Abstract 2

Crystallization processes are characterized by activated events, thus the application of enhanced sampling techniques such as metadynamics in order to study phenomena occurring at the molecular scale through molecular modelling. This paper provides an introduction to metadynamics and an overview of its applications in the context of crystal nucleation.

## 1. Introduction   

Crystals are paradigmatic examples of long-range-ordered structures obtained through a self-assembly process *via* a first-order phase transition that starts from a disordered state, such as a liquid or a gas. The initial stage of a crystallization process, nucleation, is the fundamental and yet not well understood phenomenon leading to the formation of an embryonic structure with crystal-like properties. During nucleation, atoms or molecules gather together adopting a periodic pattern reproduced in space through crystal growth (Kashchiev, 2000 ▶; Debenedetti, 1996 ▶; Agarwal & Peters, 2013 ▶). Nucleation possesses a ubiquitous character that makes it one of the most important physical phenomena in nature and industry (Doherty, 2006 ▶; Agarwal & Peters, 2013 ▶). Liquid droplets in the upper atmosphere (Koop *et al.*, 2000 ▶; Li *et al.*, 2013 ▶), biomineralization processes such as the growth of gravity-sensing devices in insects and mammals (Gago-Duport *et al.*, 2008 ▶; Weiner *et al.*, 2003 ▶; Schüler, 2002 ▶; Schüler & Frankel, 1999 ▶), as well as the production of fine chemicals and drugs (Doherty, 2006 ▶) represent only a number of selected examples among the processes crucially depending on nucleation.

An elementary understanding of nucleation can be gained with classical nucleation theory (CNT) (Kashchiev, 2000 ▶; Debenedetti, 1996 ▶; Agarwal & Peters, 2013 ▶), initially developed by Gibbs for the formation of small liquid droplets in gas. It is based on the assumption that the free energy necessary to create a nucleus of *n* particles can be divided into a favourable term, proportional to the number of particles in the nucleus, and an unfavourable term, proportional to the dividing surface between the nucleus and the solution. The free energy difference can thus be analytically expressed as 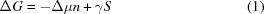
where 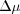 is the difference in chemical potential between the crystal and the liquid phase, *n* is the number of molecules in the crystal phase, γ is the surface tension and *S* is the surface of the nucleus. In the above expression, the surface *S* can be expressed as a term proportional to 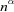 in order to have an equation depending on the size of the cluster only, where α relates to the scaling of the surface with respect to the volume of the nucleus. The simplicity of this expression is based on the assumptions that nuclei possess the same constant properties such as 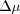 and γ regardless of the cluster size *n*. This theory has proven to be very useful for many systems, and only in recent years has an in-depth investigation of the discrepancies between CNT predictions and experiments attracted increasing interest.

Among other mechanisms and theories that have been suggested and applied, an important role is played by the *two-step* nucleation mechanism, suggested by Vekilov (2004 ▶), Kuznetsov *et al.* (1998 ▶) and Ten Wolde & Frenkel (1997 ▶). According to the *two-step* mechanism, crystal nucleation is preceded by the formation of a dense liquid phase, in which the critical nucleus emerges and starts to grow. However, no single theory has been able to completely describe the process and a comprehensive theory is still missing. Consequently, despite its importance and intensive investigation, the true nature of the nucleation process remains elusive.

The main experimental difficulties in the study of the early stages of nucleation are the small dimension of the embryo nuclei and their transient nature. Normally, quantities that are indirectly correlated with nucleation events can be measured, such as the rate, or the crystal size and shape distribution. Only for some special cases has direct investigation on the nuclei been performed (Gasser *et al.*, 2001 ▶; Wu & Yang, 2001 ▶; Liang *et al.*, 2010 ▶; Kaiser *et al.*, 2002 ▶; Harano *et al.*, 2012 ▶). For these reasons, in recent years, computational techniques such as molecular dynamics (MD) and Monte Carlo (MC) have been applied to study nucleation (Auer & Frenkel, 2001 ▶; Lechner *et al.*, 2011 ▶; Sear, 2007 ▶; Zahn, 2004 ▶; Anwar & Zahn, 2011 ▶; Peters, 2009 ▶; Harding & Duffy, 2006 ▶; Duffy & Harding, 2004 ▶).

In MD, the Newton equations of motion are numerically solved for the atoms composing the system under study. The result of the simulation is a trajectory of the system, from which kinetic, dynamics and thermodynamics quantities can be calculated. In *ab initio* MD, electronic degrees of freedom are treated explicitly, limiting the number of approximations needed. However, the time required to run these simulations increases as the third power of the number of electrons; thus only systems composed of a few hundreds of atoms are typically accessible, for a timescale of the order of a few hundreds of picoseconds (Marx & Hutter, 2009 ▶). In contrast, in classical MD, where the interactions between nuclei are treated with simplified potentials, the number of atoms can be increased up to millions and timescales of microseconds can be reached. Consequently, nucleation events are typically simulated with classical MD (Frenkel & Smit, 2001 ▶). It is worth mentioning that simulations based on *ad hoc* neural network potentials can reach time and size scales comparable to those obtained in standard MD, retaining an accuracy typical of quantum chemistry approaches, such as density functional theory (Behler & Parrinello, 2007 ▶; Sosso *et al.*, 2012 ▶, 2013 ▶).

An alternative approach to MD is to use MC simulations (Chandler, 1987 ▶; Frenkel & Smit, 2001 ▶). While MD is deterministic, MC is stochastic. In MC, a series of configurations is sequentially produced by performing random moves. The new configuration produced by a move is accepted or rejected so as to generate a desired statistical distribution. The ensemble of configurations generated in this way can be used to obtain static information on the system. In MC simulations the sequential generation of configurations does not relate to the time evolution of the system, consequently kinetic or dynamical information cannot be directly obtained from MC calculations.

The atomistic resolution of these methods makes them perfect candidates to investigate many-body problems such as crystal nucleation and growth. Unfortunately, the energy scale sampled by average fluctuations characteristic of MD or MC has an extent of a few 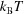 while the energy barriers associated with nucleation can be much larger. The probability of observing a nucleation event is thus negligible on a typical molecular simulation timescale.

In the last two decades a number of enhanced sampling techniques have been developed and applied to address the nucleation problem without losing the characteristic atomistic resolution (Torrie & Valleau, 1977 ▶; Dellago *et al.*, 2002 ▶; Barducci *et al.*, 2008 ▶; Laio & Parrinello, 2002 ▶; Allen *et al.*, 2009 ▶; Huber *et al.*, 1994 ▶; Marsili *et al.*, 2006 ▶). A basic idea, common to several enhanced sampling methods, is to define a set of slow degrees of freedom, or collective variables (CVs), that identify all relevant states in the phase transition. The equilibrium distribution associated with these CVs is thus suitably altered to enhance the sampling of the rare event under investigation. Typically from the post-processing of enhanced sampling simulations the unperturbed equilibrium probability distribution in the space of the CVs can be recovered (Tiwary & Parrinello, 2015 ▶), thus enabling the calculation of thermodynamic and kinetic quantities associated with the rare event. In this review we shall restrict ourselves to the application of metadynamics (Barducci *et al.*, 2008 ▶; Laio & Parrinello, 2002 ▶), to obtain information on the mechanism and thermodynamics of homogeneous nucleation.

The paper is divided into three parts. In the first part we briefly illustrate metadynamics, highlighting its features. Since this method is based on the definition of a set of order parameters to coarsen the phase space, in the second part of this paper we provide an overview of some order parameters proposed in the literature to study crystallization problems. After this methodological introduction we will illustrate a number of examples appearing in the literature. For practical reasons, we will focus on papers in which metadynamics has been applied to obtain nucleation of a crystalline material. We apologize if, in doing so, we do not mention a number of excellent papers based on other methods that have also substantially contributed to our understanding of the nucleation phenomenon and use other enhanced sampling techniques, such as umbrella sampling (Ten Wolde *et al.*, 1995 ▶, 1996*a* ▶,*b* ▶; Ten Wolde & Frenkel, 1998 ▶; Valeriani *et al.*, 2005 ▶), transition path sampling (Bolhuis, 2003 ▶; Moroni *et al.*, 2005 ▶; Zahn, 2004 ▶) or forward flux sampling (Li *et al.*, 2011 ▶).

## 2. Enhanced sampling with metadynamics   

Metadynamics belongs to the family of enhanced sampling techniques in which the probability of visiting high free energy states is increased by adding to the Hamiltonian an adaptive external potential. Such a potential, acting on some slow degrees of freedom, discourages the revisiting of states that have already been sampled and improves the exploration of the phase space. The potential is typically applied in a low-dimensional subspace defined as a function of a set of CVs, that are usually defined as continuous functions of its microscopic coordinates. The external repulsive potential is typically written as a series of Gaussian functions that are deposited during a normal MD simulation in the space of CVs as
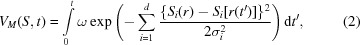
where 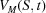 is the total bias potential deposited at time *t* in the space of the CV *S*, *d* is the dimensionality of the CVs space, 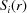 is the 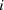
th CV, 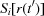 is the instantaneous value of the 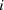
th CV, where the Gaussian contribution is centred, and ω is an energy deposition rate.

We will illustrate the effect of this potential with a simple illustrative example, in which we have a transition between two minima, separated by a high free energy barrier. In normal conditions, an MD simulation would be trapped in one of the two states, and a complete sampling of conformational space would be impossible in a practical time. However, if metadynamics is applied, the repulsive potential will act on the system pushing it out of the starting minima, over the barrier, into the second one. If the simulation is extended, the potential will eventually compensate the free energy surface, and the system will freely diffuse above the minima and the barrier, completely sampling the CVs space. At this point, an estimation of the free energy surface (FES) as a function of the set of CVs *S* can be obtained as the negative of the repulsive bias deposited during the course of the simulation
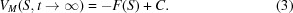
However, understanding when to terminate a standard metadynamics simulation is not trivial. In real applications, the metadynamics potential tends to overfill the underlying FES rather than compensating it. Consequently, the difference in free energy between two points does not converge to a defined value, but rather oscillates around it (Barducci *et al.*, 2008 ▶; Tiwary & Parrinello, 2015 ▶). A solution to this problem has been suggested by Barducci *et al.* (2008 ▶), by introducing a history dependence for the rate of bias deposition 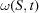 as
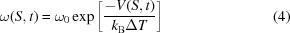
where 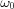 is the initial bias deposition rate, 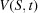 is the total bias deposited prior to time *t* and 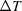 is a parameter dimensionally homogeneous to a temperature that determines the rate of decay of the Gaussian contributions. The meaning of equation (4) is that the Gaussian functions deposited along the MD trajectory are scaled with an exponential decay, that depends on the history of the system and on the position in the CV space through 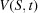. In the long time limit, the height of the Gaussian functions became negligible, and instead of compensating the FES, the well tempered (WT) meta­dynamics potential converges to the following function of the free energy 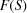


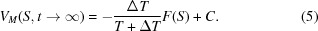
Since WT metadynamics allows one to converge the free energy and obtain a Boltzmann distribution in the space of the CV *S* (Bussi *et al.*, 2006 ▶; Dama *et al.*, 2014 ▶), this technique opens the possibility to account for the effect of the bias deposited as a function of *S* on other degrees of freedom through the formulation of reweighing algorithms (Bonomi, Barducci *et al.*, 2009 ▶; Tiwary & Parrinello, 2015 ▶). This is particularly useful, as often the degrees of freedom biased in the metadynamics calculations are not those that can be most easily connected to experiments.

It is important to highlight that the deposition of the WT metadynamics potential can be tuned in such a way that a desired probability distribution is sampled, leading to an enhancement of the fluctuations of the system along the CVs. An important application of this property is the parallel tempering (PT) well tempered ensemble simulation protocol (Bonomi & Parrinello, 2010 ▶), in which the probability of exchange between adjacent replicas, typically performed in PT (Earl & Deem, 2005 ▶), is enhanced through coupling with WT metadynamics, performed as a function of the potential energy of the system. It is important to recall that the external potential enhancing the sampling of the transition between the minima and the transition states is a function of the CVs. Consequently, the capability of metadynamics to produce a trajectory which is able to properly sample the transitions strongly depends on the choice of the CVs. Since nucleation is a many-body problem, identifying the correct CVs to generate the crystalline nuclei is far from being a simple task. Several order parameters that have been or could be used as CVs in metadynamics simulations have been proposed in the literature. Some of them are discussed in the following paragraphs.

We conclude this section by summarizing the strengths and weaknesses of the method. Metadynamics calculations are easy to set up and post-process. In contrast to other methods, it does not require an *a priori* knowledge of the CVs space, and it can be used to blindly explore the possible configurations that the system under study possesses. However, since the time that it takes to converge the FES scales exponentially with the dimension of CVs, usually no more than three CVs are used. In cases where the exploration is slow, multiple simulations or ‘walkers’ that move under the action of the same external biasing potential can be used to speed up the exploration of the FES (Raiteri *et al.*, 2006 ▶). We conclude by highlighting that in a metadynamics simulation the unperturbed dynamics and kinetics are lost. However, a framework to recover unperturbed kinetic rates has recently been suggested (Tiwary & Parrinello, 2013 ▶), and this could be used to easily relate experimental rates with metadynamics simulations.

## 3. Collective variables   

Detecting symmetry, and thus identifying molecular structures possessing a crystalline arrangement, is a task that humans can quickly and efficiently carry out based on innate pattern-recognition skills. Developing algorithms that allow one to discriminate between disordered and ordered states based on the sole Cartesian coordinates of atoms or molecules for which a molecular simulation is carried out is instead a challenging task.

The crystalline state of ensembles of molecules is typically defined on the basis of *order parameters* (OPs): functions of the Cartesian coordinates that relate a numerical value to a spatial configuration of an ensemble of atoms or molecules. An OP is typically zero for a disordered phase while it assumes characteristic nonzero values for specific spatial ordered arrangements.

Order parameters are key to the analysis of crystallization simulations as well as CVs to implement enhanced sampling protocols such as metadynamics. In the following an overview of OPs is given, with the aim of providing the flavour of the problem and some key ideas necessary to address it. Rather than compiling an exhaustive list of all the OPs developed in the literature and used in crystallization we choose to provide examples of OPs according to an incremental degree of complexity. Starting from OPs used to describe the crystal state of systems in which constituent entities possess a spherical symmetry (*i.e.* atoms or spherical particles), we will then proceed by introducing OPs suited to study crystallization in mono-component molecular systems, and then OPs used in multi-component systems such as crystals growing from solution. In this last section particular emphasis will be given to OPs used in metadynamics applications such as CVs.

### 3.1. Spherical constituent particles   

*Steinhardt parameters*. Steinhardt parameters are bond-based OPs that have been widely used to describe the crystal packing of atoms, highly symmetric molecules, or more generally objects with a geometry that can be approximated to a sphere, such as colloidal particles (Steinhardt *et al.*, 1983 ▶). Steinhardt parameters have been applied in a variety of problems in material science, spanning from the phase transition of colloidal particles to the nucleation of ice (Trudu *et al.*, 2006 ▶; Matsumoto *et al.*, 2002 ▶; Gasser *et al.*, 2001 ▶).

First of all, the definition of the Steinhardt OPs is based on a concept of bonds that does not necessarily coincide with that of a *chemical* bond. In this context the bond between two constituent particles is defined as when their distance is below a certain threshold.


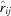
 being the unitary vector defining the direction of the bond between particles *i* and *j*, the local environment around particle *i* can be characterized by the calculation of a set of 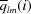


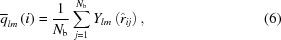
where 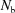 is the number of bonds in which particle *i* is involved and 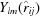 are spherical harmonics. The values of 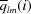 are strictly local, and thus to characterize the overall structure of an ensemble of *N* particles and obtain a global OP an average quantity has to be defined
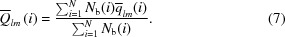
The Steinhardt OPs are defined as the first- and second-order invariant combinations of the 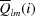


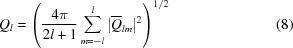
and 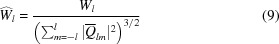
where 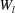 is defined as
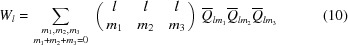
and the term in parentheses is a Wigner 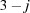 symbol.

Typical Steinhardt OPs used to characterize the crystal packing of Lennard-Jones spheres use four and six angular momenta channels. Third OPs are instead often used to characterize tetrahedral crystal environments such as the O atoms’ sublattice in ice. In cases in which rotational invariance is not relevant or it is important to promote crystallization in a specific orientation, polynomial expressions inspired by spherical harmonics but computationally cheaper can also be used as OPs, as shown by Angioletti-Uberti *et al.* (2010 ▶).

### 3.2. Molecular crystals   

In molecular crystals the simple relative position of neighbouring constituent particles is not sufficient to completely characterize the crystalline environment. In this case the complete description of the crystalline order has to account for the conformational flexibility of molecules and for the potentially wide variety of ordered arrangements. In this section we report the general framework for the construction of OPs in molecular crystals proposed by Santiso and Trout as well as some examples of OPs in which their ideas are implemented in a simplified manner. It is important to highlight that, rather than compiling an exhaustive list of all the OPs, we report some contributions that highlight different takes on the problem.

**Figure 1:**
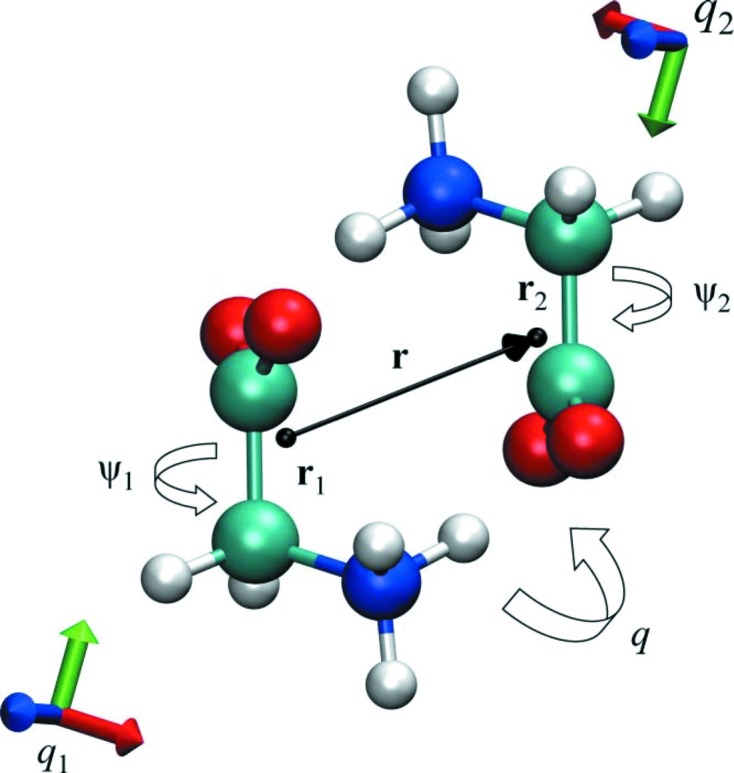
Construction of the extended pair distribution function. The vector **r** is the vector joining the centres-of-mass of molecules 1 and 2, projected onto the molecule-centred frame of molecule 1 (represented by 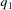). The relative orientation *q* is the quaternion that rotates the frame of molecule 1 (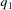) onto the frame of molecule 2 (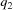), as seen from molecule 1. The internal degrees of freedom (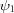 and 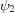) both are included in the pair distribution function. Reproduced with permission from Santiso & Trout (2011 ▶). Copyright 2011, AIP Publishing LLC.

*Describing crystal packing with an extended pair distribution function*. A generalized approach to the construction of OPs able to distinguish between the ordered and disordered states of an ensemble of molecules has recently been proposed by Santiso & Trout (2011 ▶). Santiso OPs are constructed on the notion of an extended pair correlation function, describing not only the spatial correlation of molecules’ positions, but also the spatial correlation of a set of axes describing the molecules’ relative orientation and that of a set of relevant internal degrees of freedom, characterizing the structure of the molecule. By defining ψ as a given internal configuration of a molecule, 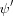 the internal configuration of its neighbour, *r* their distance and *q* their relative orientation, the generalized pair distribution function 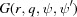 represents the probability of finding, at distance 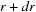, with mutual orientations 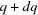 from a molecule in configuration ψ, a second molecule in configuration 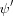. A graphical representation that helps in the rationalization of the extended pair distribution function 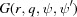 is provided in Fig. 1 ▶. Such a function, for a perfect crystal at 0 K, can be modelled as a sum over the products of δ functions as
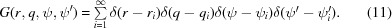
Such a sum is extended to 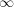, but the relevant, non-redundant terms are those characterizing the unit cell. At finite temperature the convolution of delta functions becomes a characteristic probability distribution
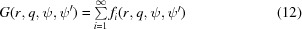
that can be approximated as the convolution of independent distributions
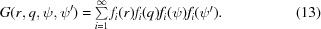
By defining specific probability distribution functions that describe 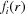, 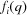, 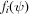, 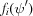 specific OPs can be built. Such OPs can be constructed in order to account for relative distances, orientations and internal configurations of organic molecules or any subset of those properties. Several examples of specific OPs are provided in the Santiso work as well as in an application to benzene melt crystallization (Santiso & Trout, 2011 ▶; Shah *et al.*, 2011 ▶), an exhaustive review of which goes beyond the scope of this paper. However, we show below how the general ideas developed by Santiso can also be found in other OPs.

*Crystalline packing of polymeric chains*. Prototypical examples of systems in which conformational flexibility dominates in the definition of the crystalline order are polymer crystals. The nucleation from polymer melts is a complex problem requiring an important computational effort. The intra-chain degree of order is the crucial aspect that has to be captured by an OP aimed at the description of the crystal state of a polymer chain. A notable example of an OP successfully addressing this issue can be found in the works of the Rutledge group (Yi *et al.*, 2013 ▶). In this work the nucleation of crystals from polymer melts is typically characterized with the 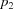 OP. The local 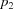 OP is computed as
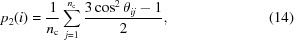
where 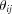 is the angle between the vector oriented as the segment joining the 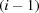-th bead in a given polymer chain with the 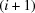-th bead, and the vector joining the 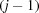-th and the 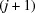-th beads. The quantity is then averaged over the 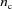 neighbouring beads lying within a predefined cutoff distance. A global OP can be constructed by averaging over all the 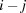 couples in the simulation box regardless of their distance.

*Asymmetry-based order parameters*. An intriguing alternative to conventional OPs used as CVs in enhanced sampling simulations or as post-processing tools comes from the biased Monte Carlo schemes employed in the works of Gavezzotti (2011 ▶, 2013 ▶). The degree of crystalline order in these works is quantified by the definition of translation and inversion asymmetry indexes 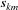. For the couple of molecules *k* and *m* these indexes are, respectively, defined as 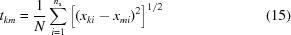 and 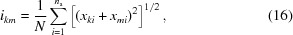
where the index *i* runs over all the 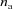 atoms in a given molecule, and 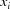 is the position of atom *i* defined with respect to a reference atom. A global, single-molecule, symmetry index is then defined for molecule *k* as 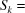 
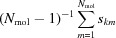
 while the total symmetry index for the whole simulation box can be obtained as 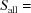 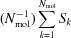. In this case, a perfect crystal has 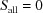 while disordered systems possess large 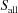 values.

### 3.3. Crystallization from solution   

When crystallization takes place from a multi-component system, such as a binary solution, local density fluctuations of solute molecules are required to nucleate a crystal. As demonstrated by Giberti *et al.* (2013 ▶), producing solution domains locally concentrated is one of the limiting steps of the process; thus using OPs that account for local density is required to describe nucleation from solution. In their work Giberti *et al.* proposed using the concentration gradient as a CV to promote NaCl nucleation in water solution. It is clear that in the case of organic molecules the concentration gradient does not completely describe the crystal order observed in a molecular system as clearly discussed and systematically assessed by Santiso & Trout (2011 ▶). To account for this effect in our recent works on urea (Salvalaglio *et al.*, 2012 ▶, 2013 ▶; Giberti *et al.*, 2015 ▶; Salvalaglio *et al.*, 2015 ▶) and 1,3,5-tris(4-bromophenyl)benzene (3BrY) (Salvalaglio *et al.*, 2014 ▶) we have developed and used an OP that accounts for both the local density of solute molecules and their relative orientation. The global OPs can be written as the sum of individual molecular contributions (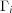) over the whole ensemble of *N* solute molecules contained in the system of interest
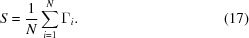
Each 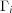 contribution depends upon the local properties of the molecular environment that embeds the molecule *i*, namely its local density, measured using the coordination number, and its orientation with respect to its neighbours. The coordination number of molecule 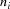 is calculated using a sum of switching functions 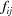


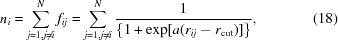
where 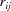 is the distance between the centre of mass of molecules *i* and *j*. The state of molecule *i* is then determined by defining another switching function, 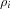, in the domain of the coordination number 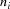 as a function of the lower coordination threshold for the crystal state 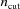


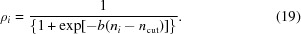
The slope of the switching functions can be tuned by choosing the parameters *a* and *b* in equations (18) and (19). To express the local order around *i*, rather than introducing a model of the generalized pair distribution function characteristic of a specific crystal structure as suggested by Santiso, an auxiliary function 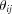 is used. This is defined in the domain of the angles between a given molecular vector describing the orientation of molecules *i* and *j* such that it is maximum for the characteristic angles observed in the crystal. We express 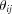 as a sum of Gaussian functions centred at specific orientation angles 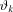 as
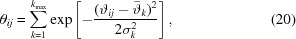
where 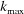 is the number of characteristic angles defining the local orientations of adjacent molecules. The choice of such a formulation introduces some flexibility in the sampling performed, *e.g.* with metadynamics. In fact, in addition to known structures, alternative local arrangements can be taken into account simply by expanding the series of 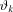 or by coupling more variables defined choosing different 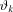 values. The local OP 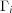 is then defined as
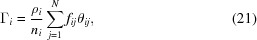
where also 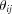 is weighted as a function of the distance between neighbours by multiplication by 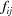 and normalized to one dividing by the coordination number 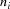. Therefore, the global OP *S* becomes
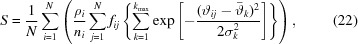
where *S* approximates the molecular fraction of constituents possessing a crystal-like coordination number that are locally ordered according to the series of reference angles 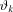.

In a recent paper, Salvalaglio *et al.* (2015 ▶) investigated the nucleation process of urea from aqueous solution using the number of crystalline nuclei and their number of constituents. These order parameters are not used frequently to study the nucleation process with enhanced sampling techniques, since they do not possess a continuous definition. However, with metadynamics it is possible to obtain an FES by reweighing the bias deposited along the MD simulation (Bonomi, Barducci *et al.*, 2009 ▶; Tiwary & Parrinello, 2015 ▶). These order parameters could be used to link more easily with experiments where the rate of nucleation, and thus the number of nuclei per unit of time, can be directly observed.

## 4. Applications   

In this part of the paper we will illustrate some examples of how metadynamics has been successfully applied to study problems related to crystallization.

**Figure 2:**
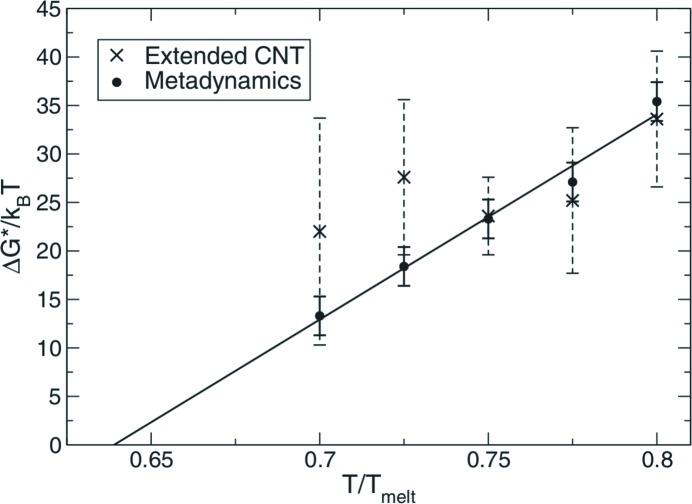
The free energy barriers as computed from metadynamics at different temperatures (circles) are compared to the work of formation of the critical nuclei and as predicted within our extended CNT model (crosses with dashed error bars). A linear fit to the metadynamics data is also shown. Reproduced with permission from Trudu *et al.* (2006 ▶). Copyright (2006) The American Physical Society.

*Lennard–Jones nucleation*. Metadynamics has been applied for the first time in the field of crystallization to study the freezing of an undercooled Lennard–Jones liquid. This system has been intensely studied over the years with brute-force MD and with other biasing methods, providing useful insight into homogeneous nucleation processes (Ten Wolde *et al.*, 1995 ▶, 1996*b* ▶; Ten Wolde & Frenkel, 1998 ▶; Bolhuis, 2003 ▶; Moroni *et al.*, 2005 ▶). The first advantage of using an enhanced sampling technique is that it is possible to study nucleation even at moderate and low undercooling, while severe undercooling has to be employed in brute-force MD or MC. This allows a better comparison with experimental data, normally sampled at low undercooling. Despite the simple structure and the absence of internal degrees of freedom, finding the correct set of CVs for this system has been a difficult task. Many authors have pointed out that the local Steinhardt parameters are the correct OPs to study the phase transition of this system, being able to identify both the f.c.c. (face-centred cubic) and the b.c.c. (body-centred cubic) arrangements emerging during the nucleation. Trudu *et al.* (2006) ▶ used the *Q* 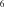 of a subset of atoms together with the potential energy of the system with plain metadynamics to drive the formation of the critical nucleus as a function of temperature. The nuclei obtained at low supercooling were for the most part non-spherical. In this regime the nucleation can be described with a modified CNT formalism that accounts for the asphericity of the nuclei. However, the mechanism changes substantially when the temperature is lowered to severe supercooling. In these conditions nucleation proceeds with the formation of more than one nucleus, that are rarely spherical and often present a fractal-like shape. The calculation of the rates as a function of temperature illustrate how at *T*/*T* 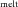 = 0.64 the nucleation rate was approaching zero. An analysis of the potential energy of the system illustrated how, in the second case, the liquid was unstable, rather than metastable. These shows support the thesis that the barrier vanishes and the surface of the critical nucleus becomes very broad and eventually diverges. The trend of the nucleation barriers as a function of the temperature, computed with metadynamics, is reported in Fig. 2 ▶.

Obtaining the FES associated with the nucleation profile from enhanced sampling techniques is of great relevance from a practical point of view. The direct calculation of the CNT FES from equation (1) with molecular simulations requires the evaluation of parameters such as the difference in chemical potential and the surface tension at the solid–liquid interface. This quantity is usually hard to evaluate, and normally only related quantities can be monitored. However, Angioletti-Uberti *et al.* developed a metadynamics framework from which surface tensions can be calculated in a simple and robust way (Angioletti-Uberti *et al.*, 2010 ▶). The key to the method is the use of a CV that is not invariant with respect to rotation, and thus enhances the formation of a solid/liquid interface oriented in a specific direction. By dividing the simulation box into two subcells and using a non-rotationally invariant degree of crystallinity in the two sub-domains as CVs, it is possible to enhance the formation of a crystal phase consistent with periodic boundary conditions. At the end of the calculation, the surface tension can thus be easily obtained as the difference between the free energy in the presence and absence of the solid/liquid interface, divided by the surface area.

**Figure 3:**
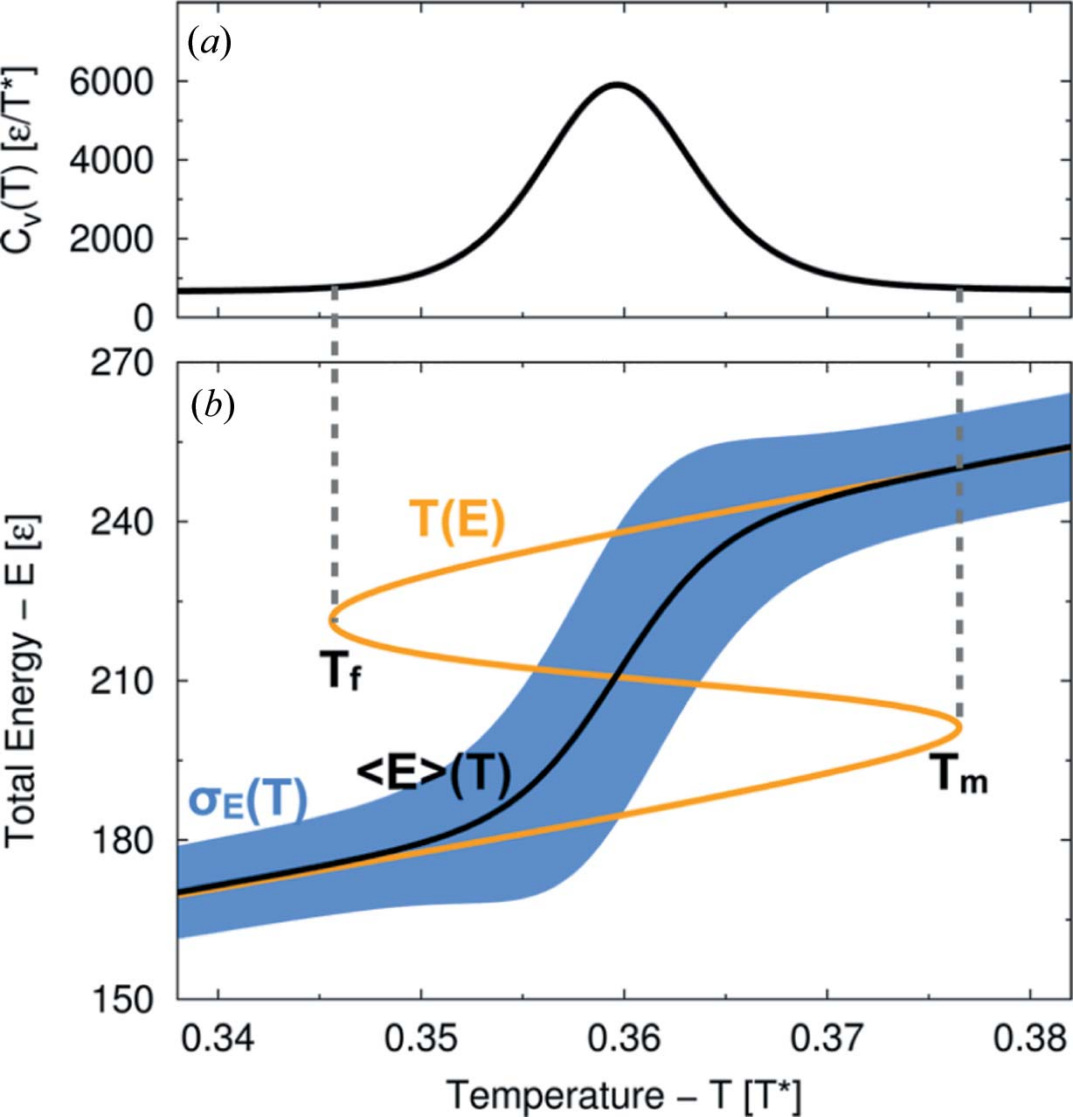
Phase-coexistence region (LJ147-1 results): (*a*) canonical specific heat Cv(*T*); (*b*) canonical caloric curve 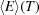 (black line), standard deviation of the total energy in the canonical ensemble 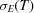 (blue shaded area) and microcanonical caloric curve 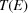 (orange line, note that for this curve the independent variable, *i.e.* the total energy *E*, is on the vertical axis). Reproduced with permission from Valsson & Parrinello (2013 ▶). Copyright (2013) American Chemical Society.

Of particular interest for its thermodynamics analysis is the paper of Valsson & Parrinello (2013 ▶). In this paper, a parallel tempering well tempered ensemble (PT-WTE) (Bonomi & Parrinello, 2010 ▶) simulation was employed to achieve the nucleation of small droplets of 147 Lennard–Jones atoms. At this small size, the process is a quasi-first-order phase transition, since first-order phase transitions are only defined for samples with an infinite number of particles. Taking advantage of the Boltzmann distribution and combining the probability density function at different temperatures, the authors were able to construct the microcanonical caloric curve 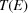 and the canonical caloric curve 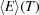 (see Fig. 3 ▶). The two curves present a difference at the phase-coexistence temperature, that should vanish when an infinite amount of particles is considered. From the canonical curve, the variance and thus the specific heat were calculated. Although the calculation was performed on a toy system, the possibility of extracting this kind of information is valuable, in particular for nanoparticles and nanorods.

As illustrated in the previous section, molecular crystals possess a higher complexity if compared to the atomic crystals discussed so far. However, there are some interesting and promising results in the literature.

*Ice nucleation*. A prime example of nucleation is the formation of ice from water. The sampling limitations associated with the activated nature of nucleation have been recently elegantly addressed by Molinero *et al.* by introducing a simple model of water (Molinero & Moore, 2009 ▶), that allows the unbiased sampling of long timescales and the characterization of rare events without the need to introduce a bias (Knott *et al.*, 2012 ▶; Moore & Molinero, 2011 ▶). Contributions in understanding ice nucleation have also been obtained using metadynamics. Quigley *et al.* enhance nucleation in ice at moderate supercooling using plain metadynamics and four OPs, 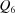, 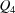, a tetrahedral OP χ (Radhakrishnan & Trout, 2003 ▶) and the potential energy of the system *U* (Quigley & Rodger, 2008*b* ▶). The CVs were chosen in order to sample the formation of both the I_c_ and I_h_ polymorphs. The nucleation of the solid phases was obtained through non-spherical embryos, typically stabilized by periodic boundary conditions. Quite often interstitial defects were included in the nucleating embryos, thus leading to an overestimation of the nucleation barrier. Despite the parameter choice, only a minor number of I_h_ water molecules were obtained, and the dominant crystal structure was found to be ice I_c_, probably due to an effect of the periodic boundary conditions.

**Figure 4:**
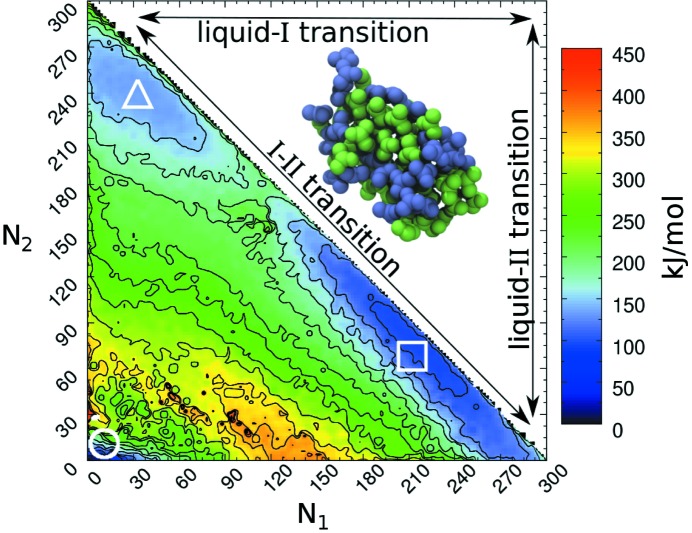
FES reweighted as a function of 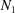 and 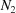, indicating the number of molecules in a form I and form II structure, respectively. Landmarks highlight the position in the CVs space of the melt (circle), polymorph I (square) and polymorph II (triangle) configurations. The transitions between the three basins of the FES described in the text are reported in the upper triangle. A nucleus in which polymorph I (blue molecules) and II (green molecules) sub-domains coexist is also shown. Reproduced with permission from Giberti *et al.* (2015 ▶). Copyright (2015) Elsevier.

*Urea nucleation from the melt*. Giberti *et al.* (2015 ▶) recently provided the formulation of a simple OP, reported in the previous paragraphs, to be employed in the description of the phase transitions in molecular systems characterized by directional orientations and few internal degrees of freedom. The OP has been applied to nucleate urea from its melt, illustrating how even simple organic molecules can exhibit complex behaviour. Systems composed of 128, 300 and 1000 urea molecules were investigated just above the melting temperature. In the 128 case, a simulation at a deep quench of 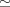 0.28 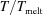 and one above the melting temperature were also carried out. Several solid/liquid phase transitions were obtained for all the systems simulated. Information on the nucleation mechanisms was obtained from the 300 and 1000 molecules, revealing a behaviour that can hardly be captured by CNT. Nuclei obtained from WT metadynamics simulations were in fact composed of molecules arranged in two different crystallographic structures. The first one is the well known urea crystal structure, composed of *head-to-tail* dimers arranged in anti-parallel chains. The second is composed of cyclic dimers. An interesting finding of this work is that a variation in the ratio between the molecules arranged as one of the two polymorphs within the nucleus corresponds to a barrierless transition, as long as the number of solid particles in the nucleus is conserved. It is important to emphasize that the process observed does not correspond to the definition of the Ostwald step rule, as the two polymorphs are not mutually exclusive. An FES obtained from the reweighing of a WT metadynamics simulation showing minima that correspond to the melt, polymorph I and polymorph II is shown in Fig. 4 ▶.

*Sodium chloride nucleation from water solution*. So far, only studies of nucleation from the molten state have been discussed. However, metadynamics has been applied in homogeneous nucleation from solution too. Giberti *et al.* (2013 ▶) investigated the homogeneous nucleation of NaCl from aqueous solution close to saturation conditions. The formation of a nucleus was driven using as CV the gradient of the concentration in the simulation box. This OP directly correlates with the presence of an interface between the NaCl ions and the water molecules. Surprisingly, in addition to the well known rock salt structure, NaCl was nucleating with another structure similar to that of wurtzite. The latter, even if less stable than the rock salt, possesses a smaller surface tension due to more favourable interactions with the solvent. This suggests a possible Ostwald step rule, where the wurtzite phase transforms into rock salt after the nucleus reaches a certain size, where the two CNT profiles cross each other.

*Calcium carbonate nucleation from water solution*. In recent years, several studies aimed at clarifying calcium carbonate (CaCO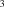) nucleation in water have been carried out using metadynamics. Biased and unbiased MD simulations have predicted a non-classical mechanism for this inorganic compound (Quigley & Rodger, 2008*a* ▶; Quigley *et al.*, 2011 ▶; Freeman *et al.*, 2010 ▶; Raiteri & Gale, 2010 ▶). In particular, several different amorphous and crystalline states, such as vaterite, aragonite and hydrated or anhydrous amorphous calcium carbonate (ACC), can be generated before forming the most stable crystalline solid (calcite). A comprehensive nucleation mechanism has still to be presented, and the relative stability of the polymorphs as well as the different nucleation rates are still debated. Consequently, meta­dynamics simulations provided interesting insight into the early stages of nucleation of CaCO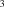. Quigley & Rodger (2008*a* ▶), using as CV the local OP *Q* 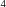, drive ACC particles of 300, 192 and 75 formula units to calcite in aqueous solution. A fine tuning of the OP allows them to identify all the possible polymorphs involved in the phase transition, ACC, vaterite, aragonite and calcite. While the larger and medium-size systems were simulated at constant pressure, the smallest was investigated at constant density too. In the 75 formula-units system, the relative stability of the ACC and the calcite structure was found to depend on the simulation set-up. In particular, at constant density the stable structure was found to be ACC, while at constant pressure calcite was the most stable form. Also the barrier between the two structures changes remarkably, from several hundreds of 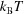 at constant density to a few tens of 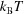 at constant pressure. For all the other particle sizes, the ACC state was found to be metastable. The two states in all the reported cases were separated by a barrier of 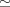 350 *k* 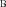 *T*, illustrating that the process needs a long time to occur, as highlighted by experimental results (Freeman *et al.*, 2010 ▶).

**Figure 5:**
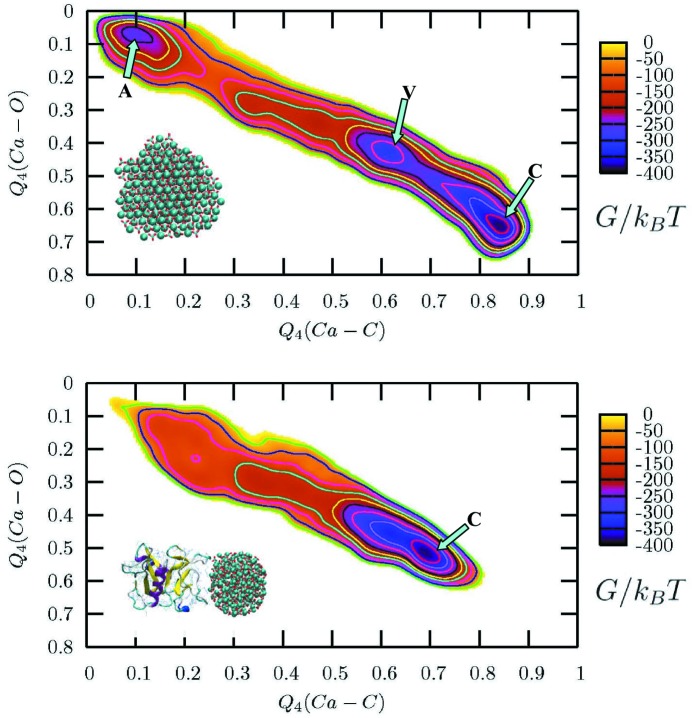
Projections of Gibbs free energy maps for a nanoparticle containing 192 units of CaCO_3_. Top: nanoparticle in water. Bottom: nanoparticle with OC-17 bound in water. The OPs used for the axes measure symmetry in the arrangement of the C or O atoms about Ca ions. The letters label minima where local order is associated with a macroscopic polymorph: A is ACC; C is calcite; V is vaterite-like. Reproduced from Freeman *et al.* (2010 ▶) with permission from Wiley.

The authors were able to identify a shallow metastable minimum between the ACC and calcite basins that could have been identified probably with vaterite, but the number of vaterite-like particles in this state was found to be too small to unambiguously assign the minimum. Aragonite was not found in the phase-transition process. The information provided in these two papers has been used by Freeman *et al.* (2010 ▶) to simulate the effect that the ovocleidin-17 (OC-17) protein would have during the nucleation of a carbonate particle. Unbiased MD simulations of the protein in contact with nanoparticles composed of 192 and 300 formula units were used to select the most relevant binding configurations. Because of the different curvature, the protein was tightly bound to the 192 formula-units particles, with two clusters of arginine, while the interaction with the 300 formula-units particle was weaker. Then, using the same set-up as Quigley & Rodger (2008*a* ▶), four metadynamics simulations for each nanoparticle were conducted. When OC-17 is present and bound to the ACC, the FES changes drastically, as displayed in Fig. 5 ▶. The barriers separating the ACC from the calcite minimum, as the intermediate vaterite-like basin, disappear. While for the small case, the protein remains bound to the CaCO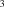 particle, in the bigger one, after the calcite crystallization, the protein desorbs from the surface. This is an intriguing fact, suggesting that this protein could in fact be part of a loop in which ACC particles are bound and transformed in calcite. The OC-17 can desorb and restart the cycle with a new ACC particle. The early stages of carbonate aggregation were studied by Tribello *et al.* (2009 ▶), using umbrella sampling and metadynamics coupled with simple MD. As observed in experiments, their simulations suggested that the formation of a hydrated phase of amorphous CaCO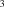 is preferred rather than the nucleation of a calcite nucleus. They illustrated how water molecules could have been kinetically trapped inside an amorphous pre-critical cluster, preventing the formation of an anhydrous crystal.

*1,3,5-Tris(4-bromophenyl)benzene nucleation from solution*. More recently, Salvalaglio *et al.* (2015 ▶) investigated with WT metadynamics the early stages of nucleation of 1,3,5-tris(4-bromophenyl)benzene (3BrY).

**Figure 6:**
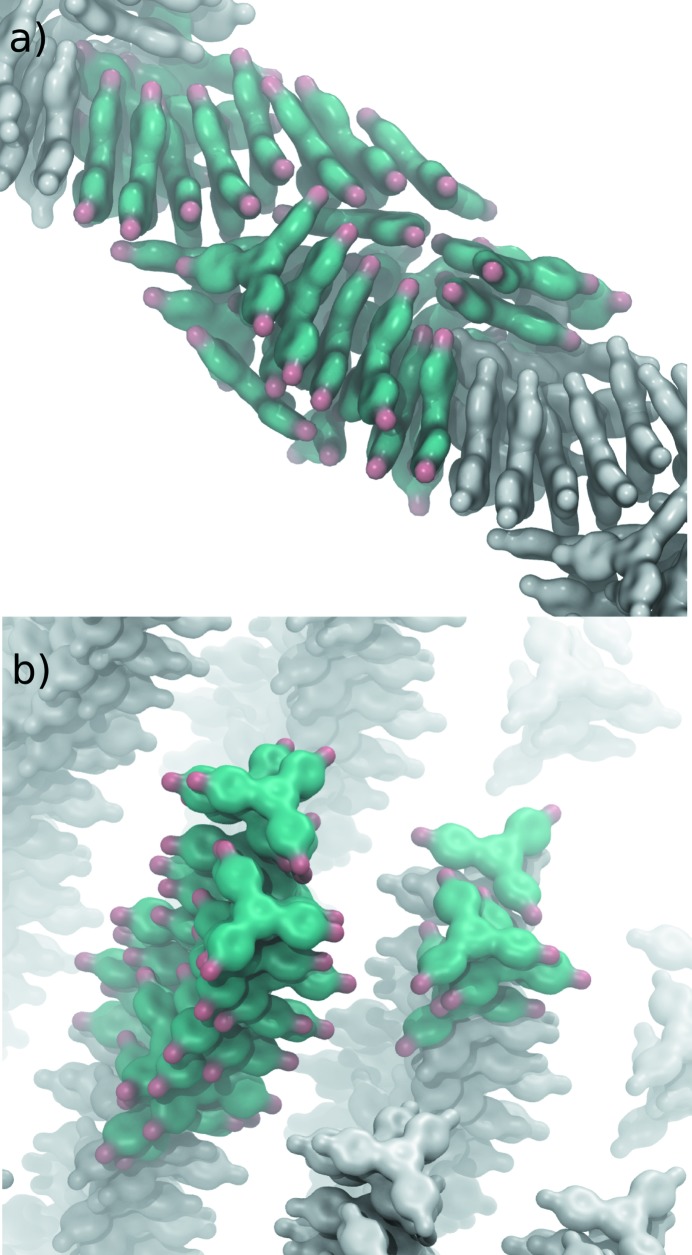
Examples of cluster structures extracted from the metadynamics trajectories in water (*a*) and methanol (*b*). Reproduced with permission from Salvalaglio *et al.* (2014 ▶).

The nucleation mechanism proposed by Harano *et al.* (2012 ▶) for this system is a two-step mechanism, where the first stage is the formation of a dense droplet of 3BrY, in which the crystal nucleates (Vekilov, 2012 ▶). The structure of the clusters obtained from WT metadynamics simulation of the 3BrY nucleation was revealed to be strongly influenced by the solvent used. In particular, long columnar structures were obtained in ethanol, while in water, where the driving force for the nucleation was stronger, 3BrY clusters are characterized by a disordered structure. Examples of such structures are reported in Fig. 6 ▶. Regardless of the solvent used, at the basis of the formation of amorphous clusters there is a strong π-stacking interaction of the 3BrY molecules that drives them to align in a crystal-like configuration. Even in small oligomers, individual molecules are arranged with the same relative orientation displayed in the crystal. However, even if similar, the structure observed in solution and the structure of the crystalline bulk are characterized by a slightly different orientation of 3BrY molecules belonging to adjacent columns, suggesting that the small size of the crystal could actually play a role in its internal structure.

## 5. Conclusions   

To conclude, in this brief review we have given a description of the ingredients necessary to investigate crystallization processes with metadynamics. First of all it is important to remark that using an enhanced sampling approach such as metadynamics is essential to compute the FES associated with the phase transition and to provide an efficient sampling of nucleation events. We highlight that the choice of the OPs to be used as collective variables is of paramount importance; we have therefore provided examples of OPs that have been or could be used in conjunction with metadynamics to explore crystallization in systems at increasing levels of complexity. Finally, a series of recent examples of crystallization problems addressed using metadynamics have been reported.
